# Metabolite profiles of *Paragliomastix luzulae* (formerly named as *Acremonium striatisporum*) KMM 4401 and its co-cultures with *Penicillium hispanicum* KMM 4689

**DOI:** 10.1007/s13659-024-00459-7

**Published:** 2024-06-18

**Authors:** Sofya S. Starnovskaya, Liliana E. Nesterenko, Roman S. Popov, Natalya N. Kirichuk, Viktoria E. Chausova, Ekaterina A. Chingizova, Artur R. Chingizov, Marina P. Isaeva, Ekaterina A. Yurchenko, Anton N. Yurchenko

**Affiliations:** https://ror.org/05t43vz03 grid.417808.2 0000 0001 1393 1398 G.B. Elyakov Pacific Institute of Bioorganic Chemistry, Far Eastern Branch of the Russian Academy of Sciences, 159 Prospect 100-Letiya Vladivostoka, Vladivostok, 690022 Russian Federation

**Keywords:** *Paragliomastix luzulae*, 28S rDNA, ITS, TEF1, Phylogeny, Identification, *Penicillium hispanicum*, Co-cultivation

## Abstract

The marine holothurian-derived fungal strain KMM 4401 has been identified as *Paragliomastix luzulae* using 28S rDNA, ITS regions and the partial *TEF1* gene sequences. The metabolite profile of the fungal culture was studied by UPLC-MS technique. The strain KMM 4401 is a source of various virescenoside-type isopimarane glycosides suggested as chemotaxonomic feature for this fungal species. Also *Px. luzulae* KMM 4401 was proposed as possible source of new bioactive secondary metabolites especially antimicrobials. Moreover, the co-cultures of *Px. luzulae* KMM 4401 with another marine fungus *Penicillium hispanicum* KMM 4689 inoculated simultaneously or after two weeks were investigated by same way. It was shown, that *P. hispanicum* KMM 4689 suppressed the production of most of *Px. luzulae* KMM 4401 metabolites. On the other hand, the co-cultivation of *P. hispanicum* KMM 4689 and *Px. luzulae* KMM 4401 resulted in increasing of production of main deoxyisoaustamide alkaloids of *P. hispanicum* KMM 4689 on 50–190%.

**Graphical Abstract:**

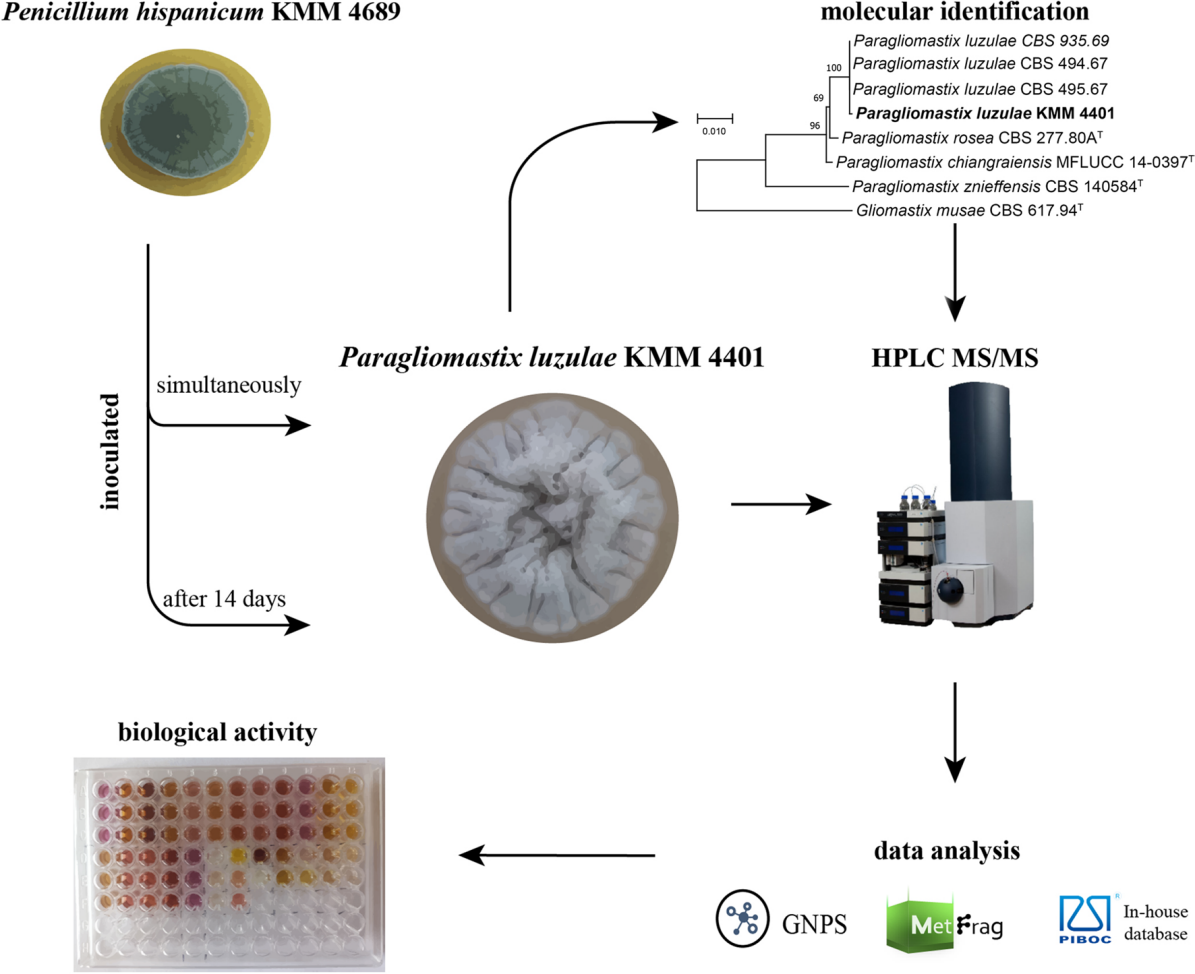

**Supplementary Information:**

The online version contains supplementary material available at 10.1007/s13659-024-00459-7.

## Introduction

The marine fungal strain KMM 4401 was isolated from the Far Eastern holothurian *Eupentacta fraudatrix* in 1995 and was identified as *Acremonium striatisporum* using morphological features [1]. Nearly thirty various diterpene glycosides virescenosides with cytotoxic activities have been isolated from this strain [2–5]. The first such diterpene glycosides, virescenosides A–H, were isolated from the terrestrial fungus *Acremonium luzulae* (preferred name *Paragliomastix luzulae* (Fuckel) L.W. Hou, L. Cai & Crous, 2023) previously incorrectly identified as *Oospora virescens* (Link) Wallr [6]. It should be noted that diterpene glycosides of this class were found only in these two fungal strains [7].

It has also been reported that *A. luzulae* can produce cyclosporin C [8]. Currently, undecapeptide cyclosporins A-Z have been isolated from various fungi [9] and were used in many studies due to their antifungal and immunosuppressive activities [10]. Cyclosporine A, known as a drug, is of the greatest interest among them, and numerous studies have been undertaken on the production of cyclosporine A under various cultivation conditions of producing fungi [11]. The authors reported the influence of stress on cyclosporin A production, but co-cultivation has not been described in this sense. Nevertheless, the high antifungal and immunosuppressive activities of cyclosporins can be "beneficial" to the fungus in its interaction with another fungus, and in that case, co-cultivation can stimulate the biosynthesis of cyclosporins or similar compounds. Indeed, fungus-fungus co-cultivation is one of the most relevant approaches for obtaining biologically active compounds [12], and many scientific groups, including ours, are working in this field [13, 14].

*Acremonium* is a highly polyphyletic genus and recently acremonium-like species have been revised to 63 genera and 14 families in *Cephalothecales*, *Glomerellales*, and *Hypocreales*, mainly in the families *Bionectriaceae*, *Plectosphaerellaceae,* and *Sarocladiaceae* and five new hypocrealean families, namely *Chrysonectriaceae*, *Neoacremoniaceae*, *Nothoacremoniaceae*, *Pseudoniessliaceae,* and *Valsonectriaceae* [15]. Based on multi-locus phylogenetic analysis, the new genus *Paragliomastix* in *Bionectriaceae* has been proposed to include *Px. chiangraiensis* (basionym *Acremonium chiangraiense*), *Px. luzulae* (basionym *Torula luzulae*), *Px. znieffensis*, and a novel species *Px. rosea* [15]. Therefore, we carried out an identification of the strain KMM 4401 using three molecular markers: 28S rRNA (large subunit ribosomal RNA), ITS and translation elongation factor EF-1 alpha (TEF1).

To determine whether strain KMM 4401 could potentially produce compounds other than those already isolated, its metabolite profile was investigated using UPLC-MS. In addition, the strain KMM 4401 was cultivated with the marine fungus *Penicillium hispanicum* KMM 4689 at two different inoculation times (simultaneously or sequentially after 14 days) to determine whether and how inoculation time affects the production of secondary metabolites. The marine strain *P. hispanicum* KMM 4689 was found to be a source of structurally interesting desoxyisoaustamide-related alkaloids [16] with promising bioactivity [17], and several studies on its fermentation have been carried out [18].

Thus, the aim of this study was to identify the marine fungal strain KMM 4401 and investigate its metabolite profile *as is* and after co-cultivation with *P. hispanicum* KMM 4689 using two different times for inoculation.

## Results

### Identification of *Paragliomastix luzulae* КMM 4401

**Fig. 1:**
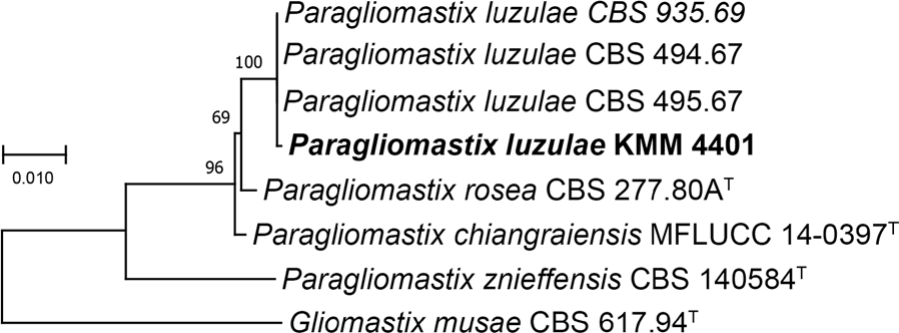
ML tree based on concatenated 28S-ITS-TEF1 nucleotide sequences showing the phylogenetic position of the strain KMM 4401 among members of the genus *Paragliomastix* from the family *Bionectriaceae*. Bootstrap values (%) correspond 1000 replications. The scale bar represents 0.01 substitutions per site

In this paper, to clarify the taxonomic position of the strain KMM 4401, we sequenced the molecular markers, such as 28S rDNA, ITS regions and the partial *TEF1* gene sequence. Approximately 950 bp fragment of the partial 28S rDNA region, about 1600 bp fragment of the ITS region, and about 1000 bp fragment of the partial *TEF1* gene sequence were successfully amplified. BLAST search showed that these sequences were 99 to 100% identical with those of the non-ex-type strains *Px. luzulae* CBS 494.67, CBS 495.67, and CBS 935.69. Phylogenetic ML tree of the concatenated 28S-ITS-*TEF1* gene sequences clearly showed that the strain KMM 4401 clusters with the strains *Px. luzulae* (Fig. 1).

### Metabolite profile of *Paragliomastix luzulae* КMM 4401

**Fig. 2:**
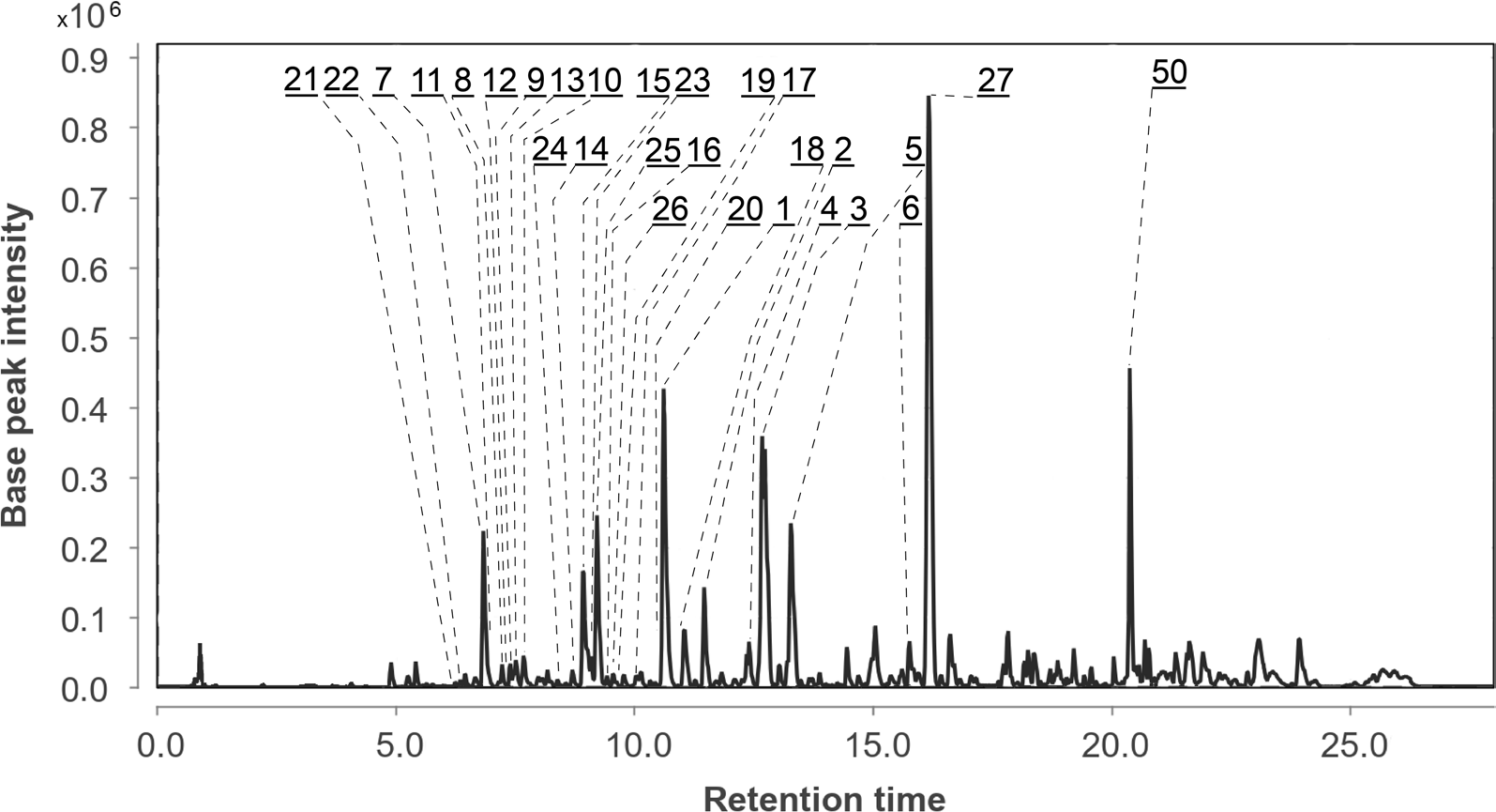
UPLC MS chromatogram of *Paragliomastix luzulae* КMM 4401 (Pl) culture

The UPLC MS chromatogram of extract of *Px. luzulae* (Pl) КMM 4401 culture is presented in Fig. 2.

**Fig. 3:**
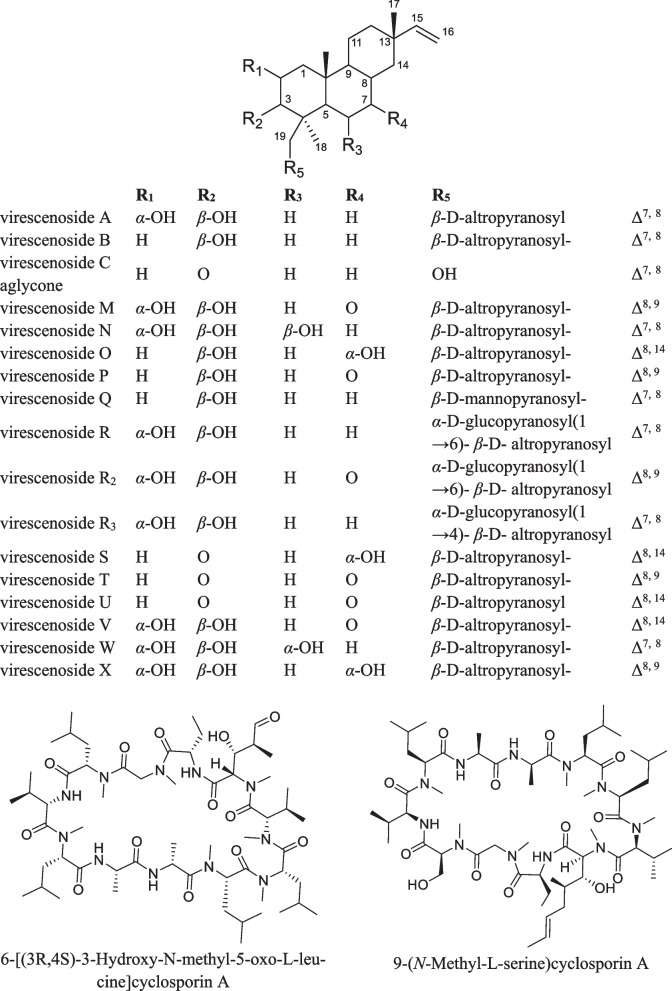
The secondary metabolites detected in *Paragliomastix luzulae* KMM 4401 monoculture

In total, 27 compounds were annotated using the in-house database or MetFrag service with PubChem database (Fig. 3, Table S1). The detailed characteristics of the identified compounds are presented in the Supplementary Materials (Table S1).

The peak #21 detected at 6.2 min at *m/z* 659.3271 corresponded to the molecular formula C_32_H_50_O_14_, the same as virescenoside R_2_, an isopimarane glycoside with a disaccharide moiety isolated early from *Px. luzulae* KMM 4401 [4]. The compound can be suggested based on an exact mass value. The peak #25 found at 9.2 min at *m/z* 479.2653 corresponded to either virescenosides T or U, as also confirmed by fragmentation under CID conditions. These two compounds have nearly identical structures, differing only in the position of the double bond in the aglycone. Moreover, four other peaks (#22, 24, 23 and 26) with similar exact mass (*m/z* 479.2618, 479.2653, 479.2618, and 479.2653) were detected at 6.4, 8.4, 9.1, and 9.6 min, respectively. All of them, have characteristic ion at *m/z* 317.2103 and 299.2005, arising from losses of altrose by in-source dissociation. Virescenosides T and U were earlier isolated from the KMM 4401 strain [19], and three additional peaks very likely corresponds to previously undescribed virescenosides, which are isomeric to virescenosides T and U.

The peak #9 found at 7.4 min at *m/z* 497.2737 corresponded to the molecular formula C_26_H_40_O_9_, which can be associated with isomeric virescenosides M and V that were isolated from the KMM 4401 strain [20, 21]. It was also suggested based on MS/MS fragmentation. In addition, three other peaks (#7, 8 and 10) with similar exact mass were detected at 6.8, 7.2 and 7.7 min, respectively. All these peaks, besides the molecular ion peak, have characteristic aglycone peaks at *m/z* 317.2103 and 335.2217, formed by in-source dissociation. Two of these peaks obviously correspond to unknown isomers of virescenosides M and V.

The peaks #11, 12, and 13 found at 6.9, 7.3, and 7.5 min at *m/z* 499.2909 corresponded to the molecular formula C_26_H_42_O_9_, which can be associated with virescenosides N [20], W and X [21] that have isomeric aglycones and were earlier described as metabolites of the KMM 4401 strain. The peak #14 found at 8.7 min at *m/z* 481.2803 corresponded to the molecular formula C_26_H_40_O_8_, which can be associated with isomeric virescenosides P [22] and S [19] that were earlier obtained from this fungal strain. Moreover, four other peaks (#15, 16, 17 and 18) were detected at 8.9, 9.4, 10.0, and 11.0 min, respectively, and have characteristic aglycone peaks at *m/z* 301.2159 and 319.2260, formed by in-source dissociation. Two of these peaks obviously correspond to unknown isomers of virescenosides N, W, and X [20, 21].

The peaks #19 and 20 detected at 9.6 min and 10.5 min at *m/z* 645.3461 and 645.3501 corresponded to the molecular formula C_32_H_52_O_13_, which can be associated with the isomeric compounds virescenoside R and R_3_. Compounds were identified based on an exact mass values and fragmentation patterns. Earlier these compounds were isolated from the KMM 4401 strain [4, 19].

The peaks #1 and 2 detected at 10.6 min and 11.5 min at *m/z* 483.2961 corresponded to the molecular formula C_26_H_42_O_8_, the same as virescenosides A [23] and O [22], respectively. Compounds were identified based on comparison of exact mass values, fragmentation patterns, and RT with whose of authentic standards.

The peak #3 detected at 12.7 min with *m/z* 467.2998 corresponded to the molecular formula C_26_H_42_O_7_, the same as virescenoside B [23]. The compound was identified based on comparison an exact mass value, MS/MS fragmentation, and RT with those of virescenoside B.

The peaks #4 and 5 found at 12.4 at *m/z* 467.2981 and 13.3 min at *m/z* 467.3015 corresponded to the molecular formula C_26_H_42_O_7_, which can be associated with virescenoside Q, known metabolite of the KMM 4401 strain [22], and another unknown isomeric virescenoside. This was suggested based on characteristic aglycone peaks at *m/z* 301.2159 and 319.2260, formed by in-source dissociation.

The peak #6 detected at 15.6 min at *m/z* 303.2325 corresponded to the molecular formula C_20_H_30_O_2_, the same as aglycone of virescenoside C [24]. The compound was identified as virescenoside C based on corresponding MS and RT data from in-house database.

The peak #27 detected at 16.2 at *m/z* 1176.7974 corresponded to the molecular formula C_59_H_105_N_11_O_13_, the same as 6-[(3*R*,4*S*)-3-hydroxy-N-methyl-5-oxo-L-leucine]cyclosporin A and 9-(*N*-Methyl-L-serine)cyclosporin A. Compound was annotated based on an exact mass value and the MetFrag service.

The peak #50 detected at 20.4 min at *m/z* 429.3350 corresponded to the molecular formula C_28_H_44_O_3_, the same as ergosterol peroxide, a usual derivative of main triterpenoid [25]. Compound was identified based on an exact mass value and RT with an in-house database.

Earlier cyclosporin A derivative was synthesized by ozonolysis of cyclosporin methyl vinyl ketone followed by reductive workup and its immunosuppressive properties was patented [26, 27]. Cyclosporin A derivative was reported in the patent as a synthetic compound [28]. So, we proposed that *Px. luzulae* КMM 4401 may be natural source of these synthetic derivatives of cyclosporin A and this strain should be promising for the isolation of this and others cyclosporin A derivatives for future investigations.

### Metabolite profile of *Paragliomastix luzulae* and *Penicillium hispanicum* co-culture with simultaneously inoculation

For this study, the culture of *Px. luzulae* KMM 4689 were seeded in flasks and *P. hispanicum* KMM 4689 was inoculated in these flasks immediately. After that the co-culture grown for 3 weeks and then was extracted for following UPLC MS analysis.

Earlier *P. hispanicum* KMM 4689 axenic culture (Ph) metabolite profile was reported [18] and in this work it was confirmed by UPLC MS technique. 25 compounds were detected in Ph extracts (Supplementary Materials, Table S1).

**Fig. 4:**
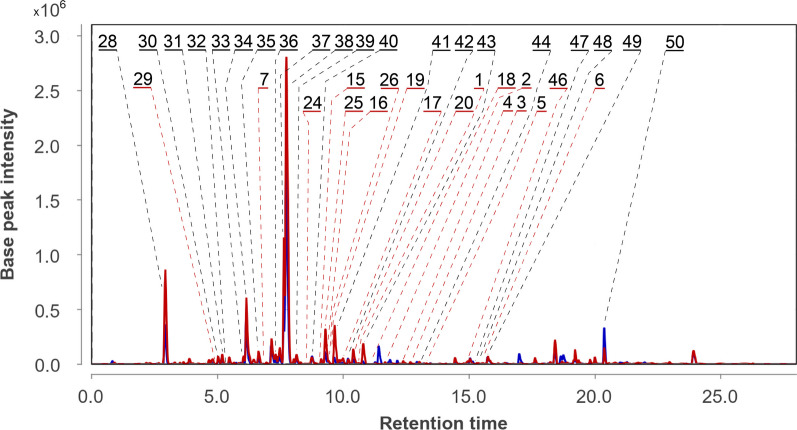
UPLC MS chromatograms of *Paragliomastix luzulae* KMM 4401 and *Penicillium hispanicum* KMM 4689 co-culture (PlPh1) (red) and *Penicillium hispanicum* KMM 4689 monoculture (Ph) (blue)

The UPLC-MS chromatogram of the extract of *Px. luzulae* KMM 4401 and *P. hispanicum* KMM 4689 simultaneously inoculated co-culture (PlPh1) in comparison with UPLC-MS chromatogram of Ph are presented in Fig. 4.

**Fig. 5:**
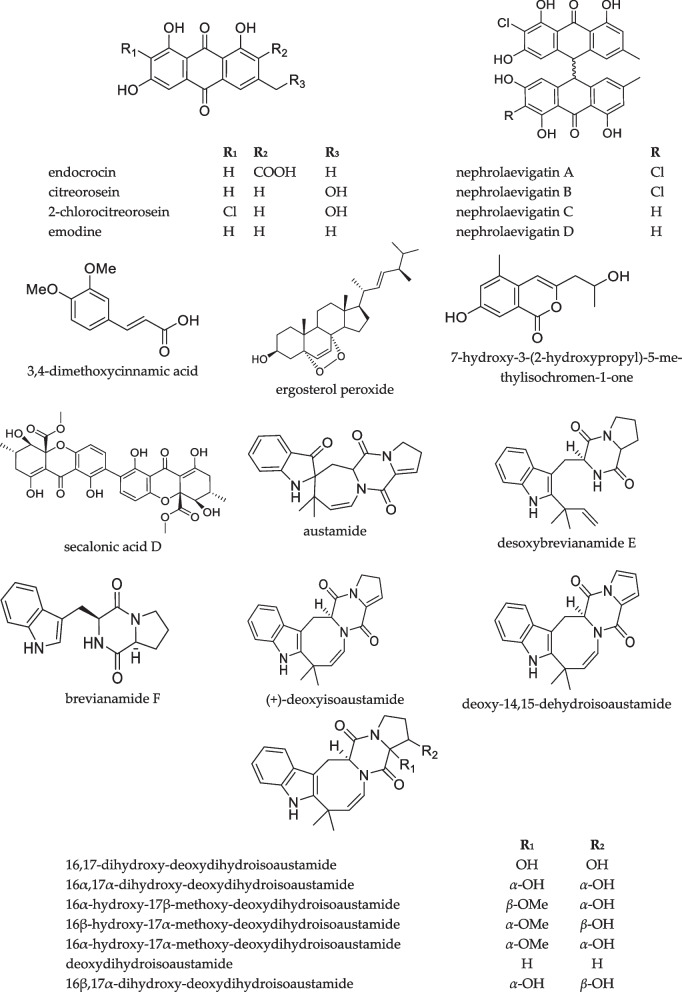
The secondary metabolites of *Penicillium hispanicum* KMM 4689 [18] detected in the co-cultures

A total of 38 compounds were successfully identified using the in-house database and GNPS (Figs. 3 and 5, Table S1). The detailed characteristics of the identified compounds are presented in the Supplementary Materials (Table S1). 17 from these peaks were corresponded to metabolites of *Px. luzulae* KMM 4401: virescenosides A (#1), B (#3), O (#2), Q/its isomer (#4 and #5), S/P/their isomers (#15–18), R/R_3_ (#19 and #20), and T/U/their isomer (#24–26), aglycone of virescenoside C (#6), as well as one from the virescenosides M/V/their isomers (#7), and cyclosporin A derivative (#27).

In addition, the compounds previously reported from axenic culture of *P. hispanicum* KMM 4689 [18] were detected: 3β-hydroxydeoxyisoaustamide (#28), 3,4-dimethoxycinnamic acid (#29), ( +)-deoxyisoaustamide (#38), 3β-hydroxydeoxyisoaustamide (#28), austamide (#32), brevianamide F (#30), 7-hydroxy-3-(2-hydroxypropyl)-5-methylisochromen-1-one (#31), 16β,17α-dihydroxy-deoxydihydroisoaustamide (#33), 16,17-dihydroxydeoxydihydroisoaustamide (#34), 16α,17α-dihydroxy-deoxydihydroisoaustamide (#35), 16α-hydroxy-17β-methoxy-deoxydihydroisoaustamide/16β-hydroxy-17α-methoxy-deoxydihydroisoaustamide/16α-hydroxy-17α-methoxy-deoxydihydroisoaustamide (#36), deoxydihydroisoaustamide (#37), endocrocin (#39), citreorosein (#40), desoxybrevianamide E (#41), 2-chlorocitreorosein (#42), deoxy-14,15-dehydroisoaustamide (#43), emodine(#44), nephrolaevigatin D (#46), nephrolaevigatin C (#47), nephrolaevigatin A (#48), and nephrolaevigatin B (#49).

### Metabolite profile of time delay co-culture of *Paragliomastix luzulae* and *Penicillium hispanicum*

Another variant of co-culture was obtained when the fungus *Paragliomastix luzulae* KMM 4401 was inoculated in the flasks and *Penicillium hispanicum* KMM 4689 culture was added in these flasks after 14 days. Then this co-culture was fermented for three weeks and then extracted for following UPLC-MS analysis.

**Fig. 6:**
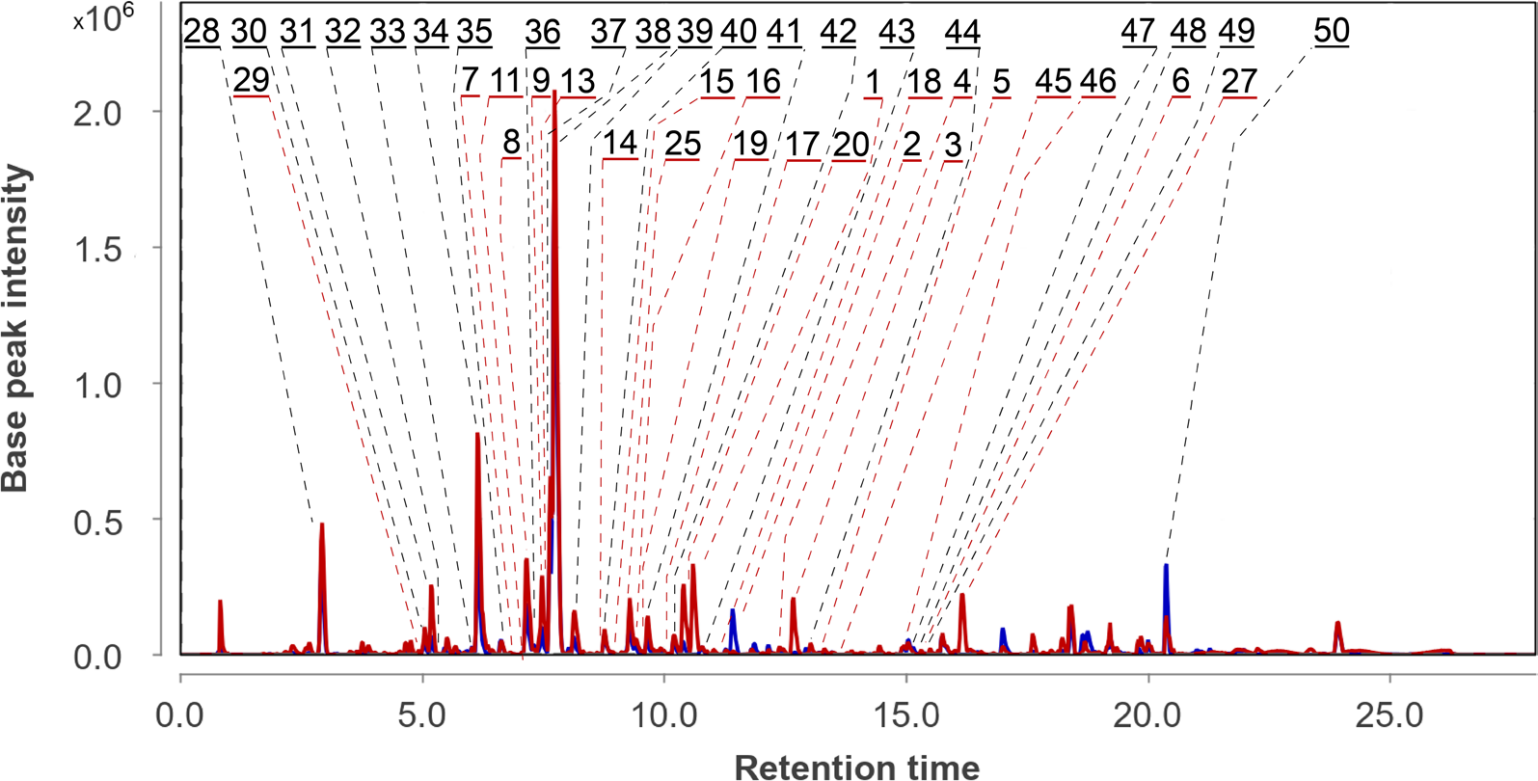
UPLC-MS chromatograms of *Penicillium hispanicum* KMM 4689 monoculture (Ph) (blue), *Paragliomastix luzulae* KMM 4401 and *Penicillium hispanicum* KMM 4689 co-culture (PlPh2) (red)

The UPLC-MS chromatogram of the extract of *Paragliomastix luzulae* KMM 4401 and *Penicillium hispanicum* KMM 4689 co-culture with time delay inoculation (PlPh2) in comparison with UPLC-MS chromatogram of Ph are presented in Fig. 6.

In total, 45 compounds were successfully identified or annotated using the in-house database and GNPS (Figs. 3 and 5, Table S1). These are 20 metabolites of *Px. luzulae* KMM 4401 including virescenosides A (#1), O (#2), B (#3), Q and its isomer (#4 and #5), virescenoside C aglycone (#6), virescenosides M/V/their isomers (#7–9), virescenoside N or W or X (#11 and #13), virescenoside S/P/their isomers (#14–18), virescenoside R/R_3_ (#19 and #20), virescenoside T or U or their isomer (#25) and cyclosporin A derivative (#27).

Moreover, 25 compounds earlier detected in axenic culture of *P. hispanicum* KMM 4689 as 3β-hydroxydeoxyisoaustamide (#28), 3,4-dimethoxycinnamic acid (#29), brevianamide F (#30), 7-hydroxy-3-(2-hydroxypropyl)-5-methylisochromen-1-one (#31), austamide (#32), 16β,17α-dihydroxy-deoxydihydroisoaustamide (#33), 16,17-dihydroxy-deoxydihydroisoaustamide (#34), 16α,17α-dihydroxy-deoxydihydroisoaustamide (#35), 16α-hydroxy-17β-methoxy-deoxydihydroisoaustamide/16β-hydroxy-17α-methoxy-deoxydihydroisoaustamide/16α-hydroxy-17α-methoxy-deoxydihydroisoaustamide (#36), deoxydihydroisoaustamide (#37), ( +)-deoxyisoaustamide (#38), endocrocin (#39), citreorosein (#40), desoxybrevianamide E (#41), 2-chlorocitreorosein (#42), deoxy-14,15-dehydroisoaustamide (#43), emodine (#44), secalonic acid D (#45), and nephrolaevigatins D (#46), C (#47), A (#48), and B (#49), as well as ergosterol peroxide (#50).

### The comparative analysis of metabolite profiles of fungal cultures

**Fig. 7:**
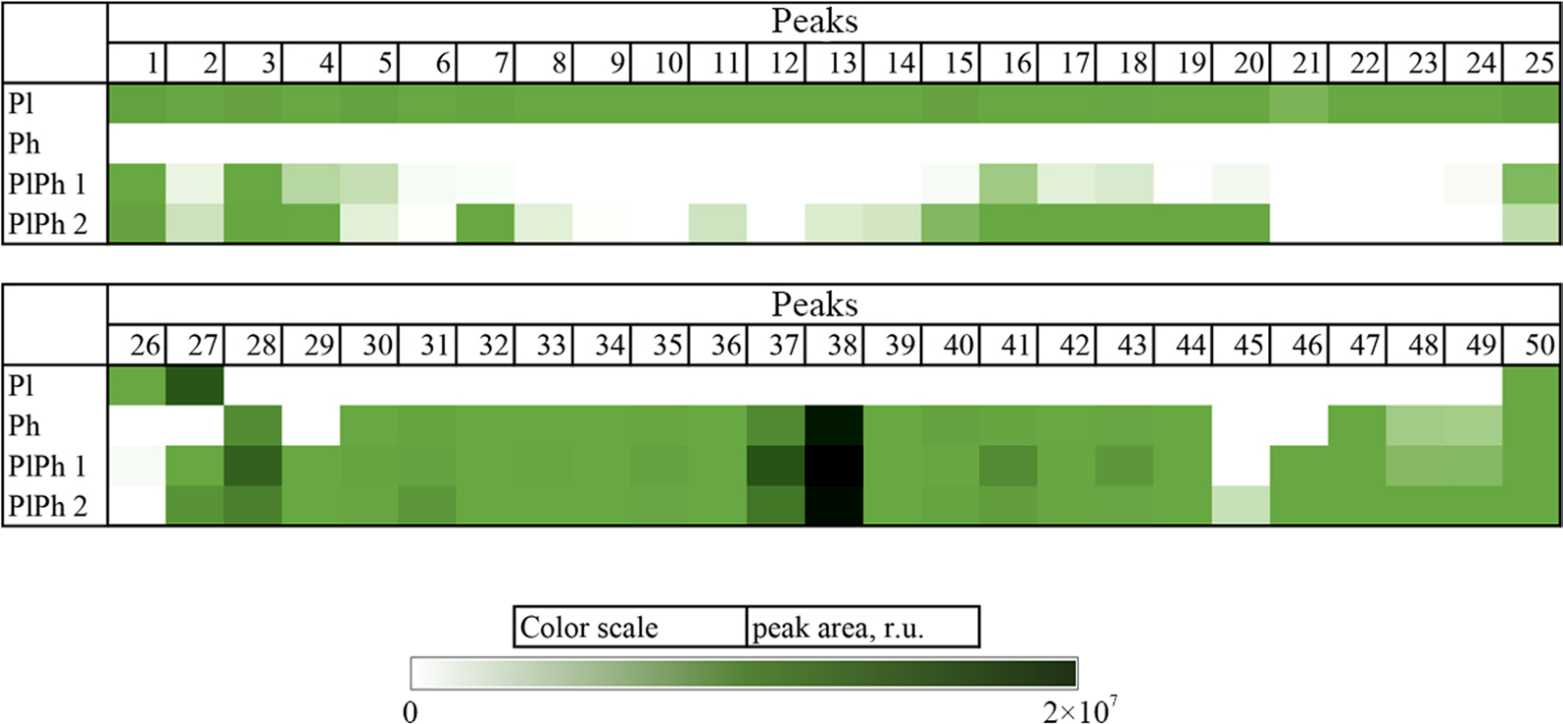
The heatmap of a related content of compounds identified in fungal extracts *Paragliomastix luzulae* KMM 4401 (Pl), *Penicillium hispanicum* KMM 4689 (Ph) and PlPh1 and PlPh2 co-cultures. Each cell presents a peak area in UPLC-MS chromatogram

The relative content of the announced compounds calculated as a ratio of the peak area to the total area of these peaks in the UPLC-MS chromatogram of Pl, Ph, PlPh1, and PlPh2 extracts was visualized in the heatmap (Fig. 7).

It was found that cyclosporin A derivative (#27) is the main component of Pl extract, but its amount dramatically decreased in both PlPh1 and PlPh2.

( +)-Deoxyisoaustamide (#38) is predominant not only in Ph extract, but in both PlPh1 and PlPh2. Moreover, the content of this alkaloid in PlPh1 extract increased by ~ 190%, and in PlPh2 extract by ~ 60% compared to the monoculture of *P. hispanicum* KMM 4689. 3β-Hydroxydeoxyisoaustamide (#28) and deoxydihydroisoaustamide (#37) are the second in content in both PlPh1 and PlPh2 extracts with an increase in concentration of ~ 170% and ~ 50% (for 3β-hydroxydeoxyisoaustamide, #28), respectively, and 68% and 12% (for deoxydihydroisoaustamide, #37). In addition, peak #46, corresponding to neprolaevigatin D, was observed only in both co-cultures, and peak #45, corresponding to secalonic acid D, was detected only in PlPh2 co-culture. At the same time, both compounds were previously described as metabolites of the axenic culture of *P. hispanicum* KMM 4689 [18].

**Fig. 8:**
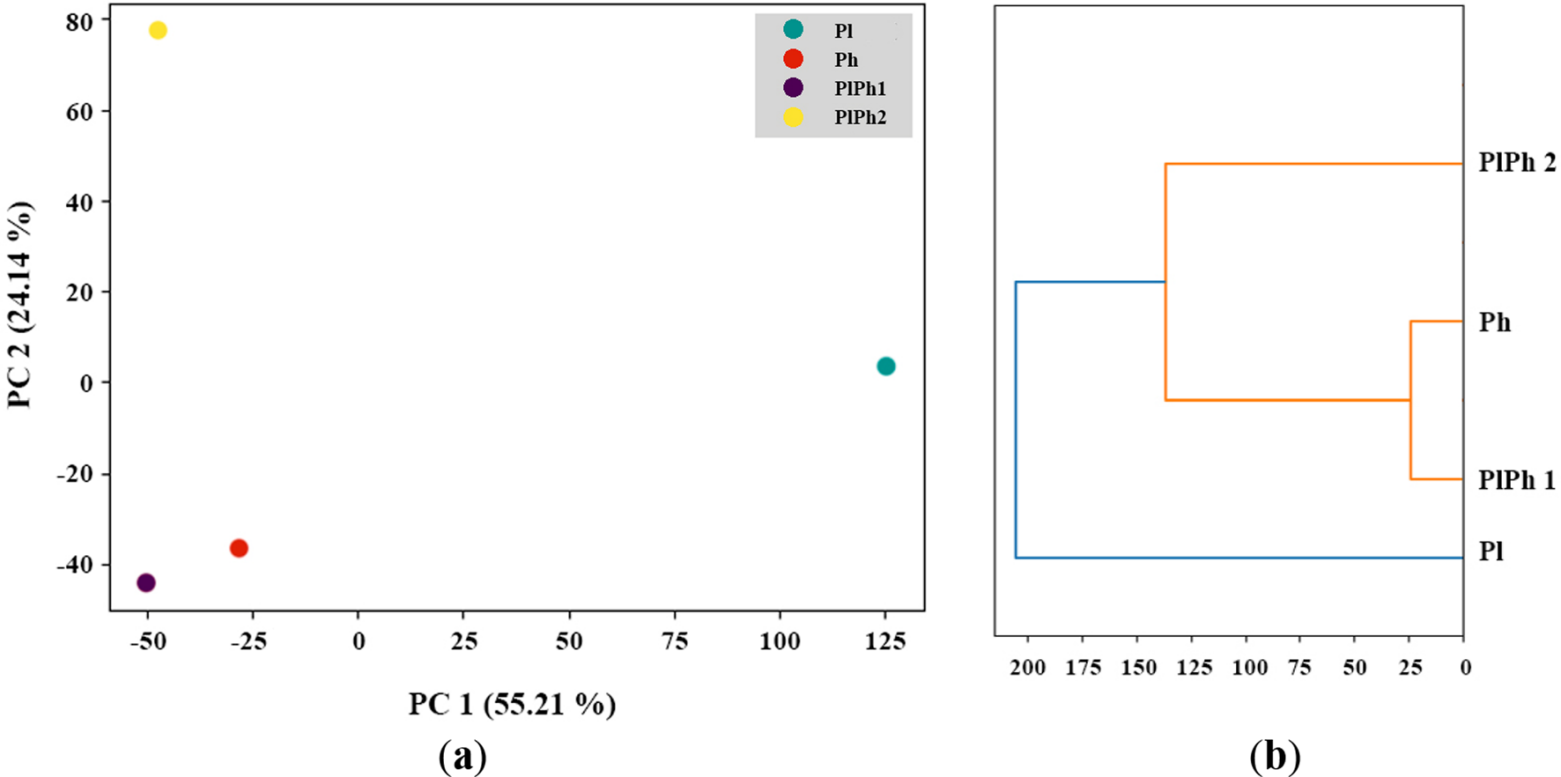
PCA plot (**a**) and dendrogram (**b**) of UPLC-MS data

The analysis of UPLC-MS data was also carried out by the principal component analysis (PCA) and both PCA plot and dendrogram are presented in Fig. 8. The PCA model determined that two principal components (PCs) were optimal for describing approximately 80% of the variation in the samples. The first PC accounted for roughly 55% of the variance, while the second PC accounted for about 24% of the variation (Fig. 8a).

The PlPh1 extract has minimal differences from the Ph extract in both components. At the same time, the PlPh2 is even more similar to the Ph in terms of the PC1 component but differs as much as possible from Ph in PC2. Both PlPh1 and PlPh2 differ significantly from the Pl extract in the PC1 component.

The dendrogram confirms the main conclusions of the PCA plot, showing the maximum similarity of PlPh1 and Ph extracts, as well as placing PlPh2 in the same cluster while Pl extract is in another cluster.

### Bioactivity of fungal extracts

The effect of Pl, Ph, PlPh1 and PlPh2 extracts on the urease activity and the growth of Gram-positive bacteria *Staphylococcus aureus,* Gram-negative bacteria *Escherichia coli* and yeast-like fungus *Candida albicans* test strains is presented in Table 1.

**Table 1:** Antimicrobial activity of extracts

Extract	The inhibition of urease activity, %	Microorganism growth inhibition, %		
		*Staphylococcus aureus*	*Escherichia coli*	*Candida albicans*
Pl	–	53.7 ± 6.6	56.4 ± 2.1	20.4 ± 1.1
Ph	–	29.1 ± 3.4	34.2 ± 0.7	–
PlPh1	–	–	–	–
PlPh2	–	18.0 ± 1.1	34.2 ± 1.9	–

All extracts were used at a concentration of 100 µg/mL. «–»—no inhibition. The data are presented as a mean ± standard error of mean. All tests were carried out in triplicates

The extract of Pl culture inhibited the growth of *S. aureus*, *E. coli* and *C. albicans* by 53.7%, 56.4%, and 20.4%, respectively. At same time, the extract of Ph culture inhibited the growth of *S. aureus* and *E. coli* by 29.1% and 34.2%, respectively, and was inactive against *C. albicans*. The extract of PlPh1 did not show any activity in this test while the extract of PlPh2 inhibited the growth of *S. aureus* and *E. coli* by 18.0% and 34.2%, respectively.

**Fig. 9:**
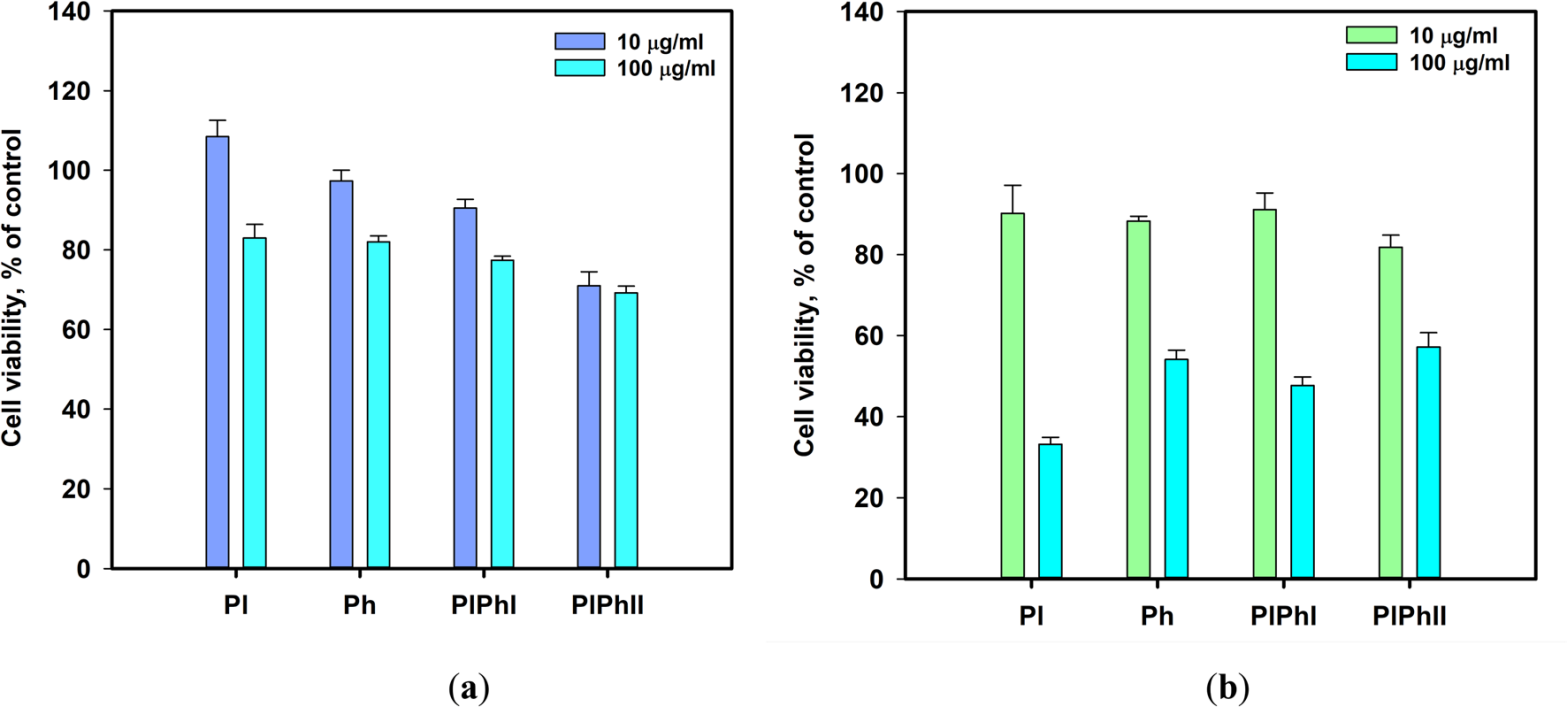
The influence of the extracts on the viability of HepG2 (**a**) and H9c2 (**b**) cells. The data are presented as a mean ± standard error of mean. All tests were carried out in triplicates

The influence of these extracts on human hepatocarcinoma HepG2 and normal rat cardiomyocytes H9c2 cells are presented in Fig. 9.

The viability of HepG2 was decrease by 17–30% when the extracts were used at a 100 µg/mL (Fig. 9a). The extracts Pl, Ph, and PlPh1 at a concentration of 10 µg/mL were nontoxic for HepG2 while PlPh2 caused the decrease of HepG2 viability by near 30%.

The toxic effect of the extracts on H9c2 cell viability was more significant (Fig. 9b). The extracts Pl, Ph, PlPh1, and PlPh2 used at a 100 µg/mL decreased the viability of H9c2 by 66.7%, 45.9%, 52.3%, and 42.8%, respectively. After dilution of the extracts by 10 times, all these decreased the viability of H9c2 by 8.9–18.2%.

DPPH radical scavenging activity of all extracts at a concentration of 100 µg/mL was measured. The extracts Pl, Ph, PlPh1, and PlPh2 scavenged 48.5%, 16.0%, 34.2%, and 49.7% of DPPH radicals.

## Discussion

Thus, fungal strain KMM4401 identified earlier as *A. striatisporum* only by morphologic features, has now been re-identified as *Px. luzulae* using molecular genetic approach. As noted in the Introduction, *Acremonium* is a taxonomically difficult and highly polyphyletic group of ascomycetes. Combined the phylogenetic trees analysis with morphological characteristics, host and ecological analyses, acremonium-like species were recently combined to 63 genera, and 14 families including five new [15]. According to this new taxonomic system, *Paragliomastix luzulae* is actual name for synonyms *Acremonium luzulae*, *Sagrahamala luzulae*, and *Gliomastix luzulae* [29]*.*

The detail investigation of metabolite profile of marine fungus *Px. luzulae* KMM 4401 isolated from holothurian was carried out by UPLC-MS. This made it clear that the secondary metabolites of this strain are more diverse than was understood after a series of publications on isolation and structure determination of its low molecular weight compounds. To date, it is known about 38 isopimarane diterpene glycosides, virescenosides, previously isolated from the strain KMM 4401, but the new data suggest that at least nine other related compounds can be still isolated. It should be note that totally only 118 diterpene glycosides were isolated from various fungi and only 59 from them contain isopimarane aglycones [30]. So, the *Px. luzulae* KMM 4401 strain produces the third part of all known fungal diterpene glycosides, and our observations made this fungus very interesting for future investigation. The re-identification of the KMM 4401 strain as *Px. luzulae* and the fact that virescenosides were isolated only from fungi of this species suggests that isopimarane glycosides of this type are a chemotaxonomic marker for these fungi.

Moreover, UPLC-MS data showed that this marine fungus may produce cyclosporin A related peptides which earlier were not reported for this strain. The bioactivity of *Px. luzulae* KMM 4401 extract further confirms the promise of its further study, since it has been established its significant antimicrobial activity, which was not reported for earlier isolated compounds.

Co-cultivation of fungal strains is a common way to influence the production of their secondary metabolites. In this case, we chose the *P. hispanicum* KMM 4689 as the second strain for co-cultivation with *Px. luzulae* KMM 4401*.*

Earlier it was showed that *P. hispanicum* KMM 4689 reacted to changes in the composition of the environment by changing the production of secondary metabolites [18], and in a co-culture with marine isolate of *Aspergillus fumigatus* it produced new metabolites [31]. In present work, *P. hispanicum* KMM 4689 has some ability to suppress the production of the secondary metabolites of *Px. luzulae* KMM 4401 that was observed both in UPLC-MS data and antimicrobial activity of extracts. Despite some increase of cytotoxicity of co-cultures extracts toward human hepatocarcinoma HepG2 cells were detected, it does not appear that any new low molecular weight compounds announced in significant quantities in investigated fungal co-cultures. ( +)-Deoxyisoaustamide, 3β-hydroxydeoxyisoaustamide and deoxydihydroisoaustamide from *P. hispanicum* KMM 4689 were main components in *P. hispanicum* KMM 4689 and *Px. luzulae* KMM 4401 co-cultures extracts, but it has not previously been established any their biological activity which can explain the suppress of *Px. luzulae* KMM 4401 in studied co-cultures. But it is likely that they play some role in this, and it may be aim for future investigation.

Another implication of this work is that the timing of inoculation in co-cultivation of fungi in lab matters. It was observed that *P. hispanicum* KMM 4689 suppressed the production of secondary metabolites of *Px. luzulae* KMM 4401 during co-cultivation of these fungi with simultaneous inoculation. At the same time, the production of some *P. hispanicum* alkaloids increased significantly. When *P. hispanicum* KMM 4689 was inoculated two weeks later, the production of metabolites of *Px. luzulae* KMM 4401 increased, but the production of deoxyisoaustamide alkaloids was less than in case of simultaneous inoculation. Deoxyisoaustamide alkaloids have promising biological activities [32, 33] and co-cultivation of *P. hispanicum* KMM 4689 and *Px. luzulae* KMM 4401 with simultaneous inoculation can be one of the ways to obtain them in large quantities for future investigations of its bioactivities and semi-synthesis of unique minor derivatives.

## Conclusions

The marine holothurian-derived fungal strain KMM 4401 was identified as *Px. luzulae* using 28S rDNA, ITS regions and the partial *TEF1* gene sequence. This fungus was confirmed as a source of various glycosides as well as possible source of new bioactive secondary metabolites especially antimicrobials. The co-cultivation of *Px. luzulae* KMM 4401 with another marine fungus *P. hispanicum* KMM 4689 resulted in the reducing of the secondary metabolites’ production by *Px. luzulae* fungus. The co-cultivation of *P. hispanicum* KMM 4689 and *Px. luzulae* KMM 4401 with simultaneous inoculation can be considered as a promising way to obtain the new metabolites in large quantities for future investigations.

## Materials and methods

### General

Microscopic examination and photography of fungal cultures were performed with an Olympus CX41 microscope fitted with an Olympus SC30 digital camera. Detailed examination of ornamentation of the fungal conidia was performed using scanning electron microscopy (SEM) EVO 40.

Low-pressure liquid column chromatography was performed using a Gel ODS-A (12 nm, S—75 um, YMC Co., Ishikawa, Japan). Plates precoated with Si gel (5–17 μm, 4.5 cm × 6.0 cm, Imid Ltd., Krasnodar, Russia) were used for thin-layer chromatography.

### Fungal strains

The fungal strain KMM 4401 was isolated from superficial mycobiota of the sea cucumber *Eupentacta fraudatrix* collected in the Kitovoye Rebro Bay (the Sea of Japan) and identified based on morphological features as *Acremonium striatisporum* [20]. The fungal strain KMM 4689 was isolated from unidentified soft coral collected near Con Co Island (the South China Sea, Vietnam) and identified based on molecular features as *Penicillium hispanicum* [18].

All used fungal strains are stored in the Collection of Marine Microorganisms (PIBOC FEB RAS, Vladivostok, Russia). The strain *Penicillium hispanicum* KMM 4689 also stored in the Collection of Nhatrang Institute of Technology Research and Applications under the code VO49-30.5.

### DNA extraction and amplification

Genomic DNA was isolated from fungal mycelia (mycelium) grown on MEA (malt extract agar) at 25 °C for seven days, using the MagJET Plant Genomic DNA Kit (Thermo Fisher Scientific, Waltham, MA, USA), according to the manufacturer’s protocol. PCR was conducted using GoTaq Flexi DNA Polymerase (Promega, Madison, WI, USA). The 28S rDNA region was amplified using the standard primer pair LROR and LR5 [34]. The reaction profile was 95 °C for 300 s, 35 cycles of 95 °C for 30 s, 55 °C for 45 s, and 72 °C for 120 s, and finally 72 °C for 300 s. For amplification of the internal transcribed spacer region (ITS) were used the primer pair ITSpr1 (5′-GCGTTGATATACGTCCCTGCC-3′) [35] and ITSpr7 (previously named as D3B*-R) (5′-ACTTCGGAGGGAACCAGCTAC-3′) [36]. The reaction profile was 95 °C for 300 s, 35 cycles of 95 °C for 20 s, 55 °C for 20 s, and 72 °C for 120 s, and finally 72 °C for 300 s. For amplification of the TEF1 gene the standard primer pair EF1-983F and EF1-2218R was used [37]. The reaction profile was 95 °C for 300 s, 35 cycles of 95 °C for 30 s, 50 °C for 45 s, and 72 °C for 90 s, and finally 72 °C for 300 s. The amplified 28S rDNA, ITS and TEF1 fragments were purified and sequenced as described in [38]. Gene sequences were deposited in GenBank under accession numbers OR650021 for 28S, OR650019 for ITS and OR672062 for TEF1 (Table 2).

**Table 2:** The strains of the species used in multi-locus phylogenetic analysis and GenBank accession numbers

Species	Strain number	GenBank accession number		
		LSU	ITS	*TEF1*
*Paragliomastix chiangraiensis*	MFLUCC 14-0397^T^	NG_068918	NR_186938	–
*Paragliomastix rosea*	CBS 277.80A^T^	OQ055673	OQ429775	OQ471103
*Paragliomastix znieffensis*	CBS 140584^T^	NG_058205	OQ429776	OQ471104
*Paragliomastix luzulae*	CBS 494.67	MH870757	MH859040	OQ471101
*Paragliomastix luzulae*	CBS 495.67	HQ232058	OQ429773	–
*Paragliomastix luzulae*	CBS 935.69	MH871267	MH859486	OQ471102
*Paragliomastix luzulae*	KMM 4401	OR650021	OR650019	OR672062
*Gliomastix musae*	CBS 617.94^ T^	OQ055523	OQ429616	OQ470926

^T^Ex-type strain

### Phylogenetic analysis

The 28S rDNA, ITS and TEF1 nucleotide sequences of the fungal strain KMM 4401 and members of the genus *Paragliomastix* of the family *Bionectriaceae* were aligned by MEGA X software version 11.0.9 [39] using Clustal W algorithm. The ex-type homologs and non-ex-type homologs were searched in the GenBank database (http://ncbi.nlm.nih.gov) using the BLASTN algorithm (http://www.ncbi.nlm.nih.gov/BLAST, accessed on 02 October 2023). The phylogenetic analysis was conduct using MEGA X software [39]. The 28S rDNA, ITS regions and partial TEF1 gene sequences were concatenated into one alignment. Phylogenetic tree was constructed according to the Maximum Likelihood (ML) algorithm based on the Tamura 1992 model [40]. The tree topology was evaluated by 1000 bootstrap replicates. The *Gliomastix musae* CBS 617.94^T^ was used in the phylogenetic analysis as outgroup (Table 2).

### Cultivation of fungi

Before co-cultivation, fungal strains* Px. luzulae* KMM 4401 и *P. hispanicum* KMM 4689 were grown in test tubes on slanted wort agar in sea water for 14 days at 22 °C. Subsequently, the resulting fungal cultures were used for cultivation on rice medium. Sowing for joint growth was carried out in two variants: in the first case, inocula of 2-week-old fungal strains were introduced into flasks with rice medium simultaneously, in the second case—with a difference of two weeks, considering the different growth rates of the studied crops. In the second case, the inoculum of *P. luzulae* was first added to the medium and cultivated at 22 °C for two weeks. After 14 days, an inoculum of *P. hispanicum* was added to the same flask. Co-cultivation of the strains was carried out at room temperature for 21 days.

### Extraction of fungal cultures

Each fungal culture with medium was extracted with EtOAc (100 mL) and then evaporated *in vacuo* to yield a crude extract (Table 3). Then each extract was dissolved in methanol and passed through column with C_18_-SiO_2_ (YMC Gel ODS-A). The masses of purified extracts are presented in Table 3.

**Table 3:** Amounts of the extracts of the fungal cultures

Fungal culture	Sample code	Mass of crude extract, mg
*Paragliomastix luzulae* KMM 4401	Pl	38.3
*Penicillium hispanicum* KMM 4689	Ph	233.1
*Co-culture of Paragliomastix luzulae* KMM 4401 and *Penicillium hispanicum* KMM 4689 (simultaneous inoculation)	PlPh1	89.1
*Co-culture of Paragliomastix luzulae* KMM 4401 and *Penicillium hispanicum* KMM 4689 (inoculation of *P.hispanicum* after 14 days)	PlPh2	123.1

#### UPLC-MS analysis of fungal extracts

UPLC-MS analysis was performed using a Bruker Elute UPLC chromatograph (Bruker Daltonics, Bremen, Germany) connected to a Bruker Impact II Q-TOF mass spectrometer (Bruker Daltonics, Bremen, Germany). An InfinityLab Poroshell 120 SB-C18 column (2.1 × 150 mm, 2.7 μm, Agilent Technologies, Santa Clara, CA, USA) was used for chromatographic separation. The detailed description of chromatographic separation and mass spectrometric detection were reported earlier [18].

#### UPLC-Q-TOF data analysis

UPLC-Q-TOF data were converted from Bruker “.d” formatting to “.mzXML” using MSConvert 3.0 (part of ProteoWizard 3.0 package, Palo Alto, CA, USA) [41], and further processing was performed using MZMine (version 2.53) [42] as described previously [18].

In addition, the identification of some metabolites was performed by comparing of experimental MS/MS spectra with compounds from the PubChem database using in-silica fragmentation by MetFrag service [43]. Metabolite dereplication was also carried out with an in-house MS/MS spectral library [18].

### Principal component analysis ((PCA)

PCA analysis, a hierarchical dendrogram, and visualization of the resulting graphs were performed using the “google colab” web resource based on Python 3.8 using Pandas, Seaborn, and Matplotlib libraries. Below is a link to the notepad with the code used in the analysis:https://drive.google.com/drive/folders/1gTZMlPZXXbFH3UBE5FjaTMzETgmqmYjr?usp=sharing

### Bioassays

#### Urease inhibition assay

The urease inhibitory activity was estimated by determining ammonia production using the indophenol method. Urease from *Canavalia ensiformis* (final concentration 1U) was used in this test. A reaction mixture consisting of 25 µL enzyme solution and 5 µL of extracts (100.0 µg/mL final concentration) was preincubated at 37 °C for 60 min in 96-well plates. Then, 55 µL of phosphate buffer solution with 100 µM urea was added to each well and incubated at 37 °C for 10 min. Then, 45 µL of phenol reagent (1% w/v phenol and 0.005% w/v sodium nitroprusside) and 70 µL of alkali reagent (0.5% w/v NaOH and 0.1% active chloride NaClO) were added to each well. The pH was maintained at 7.3–7.5 in all assays. DMSO 5% was used as a positive control. The absorbance was measured after 50 min at 630 nm using a MultiskanFS microplate reader (Thermo Scientific Inc., Beverly, MA, USA).

#### Antimicrobial activity

The Gram-positive bacteria *Staphylococcus aureus* ATCC 21027, Gram-negative bacteria *Escherichia coli* VKPM (B-7935) and yeast-like fungi *Candida albicans* KMM 455 strains were fermented on solid medium Mueller Hinton broth with agar (16.0 g/L) in a Petri dish at 37 °C for 24 h.

The assays were performed in 96-well microplates in appropriate Mueller Hinton broth. The test strains’ suspensions (90 µl, 10^6^ CFU/mL) were added in each well and then 10 µL of the extract diluted at concentrations from 12.5 µg/mL to 100.0 µg/mL using two-fold dilution was added. The concentration of vehicle (DMSO) was less than 1%. The antibiotic gentamicin and the antifungal agent nitrofungin were used as positive controls at 1 mg/mL. Moreover, DMSO (1% in PBS) served as a negative control. The plates were incubated for 18 h at 37 °C, and the OD_620_ was measured using a Multiskan FS spectrophotometer (Thermo Scientific Inc., Beverly, MA, USA). The inhibition of test strains’ growth was calculated as % = (OD_control_ – OD_sample_)/OD_control_*100% [44].

#### DPPH radical scavenger assay

The methanol solution of DPPH (Sigma-Aldrich, Steinheim, Germany) at a concentration of 7.5 × 10^−3^ M was used. The dry extracts were dissolved in MeOH at a concentration of 125 µg/mL, and the final concentration in reactive mixture was 100 µg/mL. The optical density of the mixture after 30 min was detected at 520 nm with a microplate reader MultiscanFC (ThermoScientific, USA). The radical scavenging activities of the extracts at 100 µg/mL were calculated as % of the control (MeOH).

#### Cell culture

The human hepatocarcinoma HepG2 HB-8065 ™cells were purchased from ATCC (Manassas, VA, USA). The rat cardiomyocytes H9c2 cells were kindly provided by Prof. Dr. Gunhild von Amsberg from Martini-Klinik Prostate Cancer Center, University Hospital Hamburg-Eppendorf, Hamburg, Germany. The cells were cultured in DMEM with 10% of fetal bovine serum and 1% of penicillin/streptomycin (BioloT, St. Petersburg, Russia). For experiments HepG2 and H9c2 cells were seeded at concentrations of 5 × 10^3^ cell/well and 3 × 10^3^ cell/well, respectively, and the experiments were started after 24 h.

#### Cell viability assay

The cells were treated with extracts at a concentration of 10 µg/mL and 100 µg/mL for 24 h, and the viability of cells was measured using an MTT (3-(4,5-dimethylthiazol-2-yl)-2,5-diphenyltetrazolium bromide) assay according to the manufacturer’s instructions (Sigma-Aldrich, St.-Louis, MO, USA). The results are presented as a percentage of control data.

#### Statistical data evaluation

All bioassays’ data were obtained in three independent replicates, and calculated values are expressed as a mean ± standard error mean (SEM) using SigmaPlot 14.0 (Systat Software Inc., San Jose, CA, USA).

### Supplementary Information

Supplementary Material 1.

## Data Availability

The original data presented in the study are included in the article/Supplementary Material; further inquiries can be directed to the corresponding author.
