# Supplementary material for: Metabolite profiles of Paragliomastix luzulae (formerly named as Acremonium striatisporum) KMM 4401 and its co-cultures with Penicillium hispanicum KMM 4689
Source: Nat Prod Bioprospect. 2024 Jun 18;14(1):38. doi: 10.1007/s13659-024-00459-7 (PMC11182996; doi:10.1007/s13659-024-00459-7)
Supplement: Supplementary file 1 — Supplementary Material 1. [file 13659_2024_459_MOESM1_ESM.pdf]

Supplementary Materials

Metabolite Profiles of *Paragliomastix luzulae* (Formerly Named as *Acremonium striatisporum*) KMM 4401 and Its Co-cultures with *Penicillium hispanicum* KMM 4689

Sofya S. Starnovskaya, Liliana E. Nesterenko, Roman S. Popov, Natalya N. Kirichuk, Viktoria E. Chausova, Ekaterina A. Chingizova, Artur R. Chingizov, Marina P. Isaeva, Ekaterina A. Yurchenko and Anton N. Yurchenko

Table S1. Secondary metabolites annotated in studied fungal extracts

Paragliomastix luzulae KMM 4401

№	Culture	RT	m/z	Suggested structure and name	Exact mass	Mass error
1	Pl<br>PlPh1<br>PlPh2	10.6	483.2961<br>[M+H]+	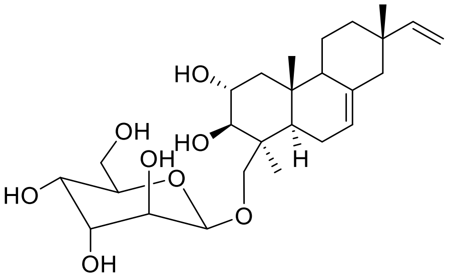<br>virescenoside A	483.2953	1.7
2	Pl<br>PlPh1<br>PlPh2	11.5	483.2961<br>[M+H]+	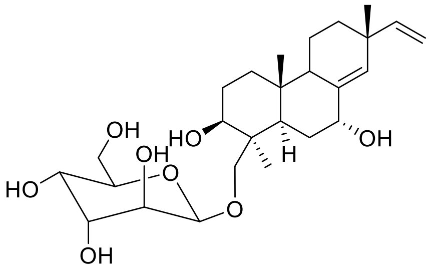<br>virescenoside O		
3	Pl<br>PlPh1<br>PlPh2	12.7	467.2998<br>[M+H]+	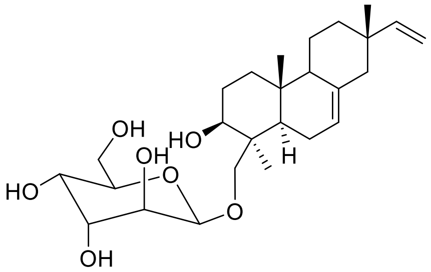<br>virescenoside B	467.3003	–1.1
4	Pl<br>PlPh1<br>PlPh2	12.4	467.2981<br>[M+H]+	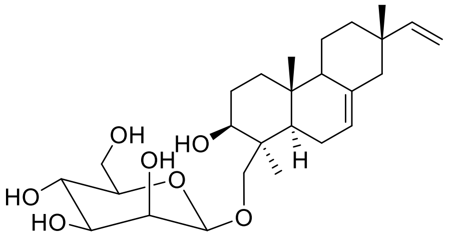<br>virescenoside Q<br>or<br>its isomer		-4.7
5	Pl<br>PlPh1<br>PlPh2	13.3	467.3015<br>[M+H]+			2.6
6	Pl<br>PlPh1<br>PlPh2	15.6	303.2325<br>[M+H]+	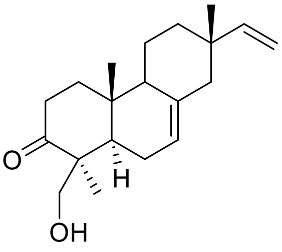<br>virescenoside C aglycone	303.2319	2.0

7	Pl<br>PlPh1<br>PlPh2	6.8	497.2737<br>$[M+H]^+$	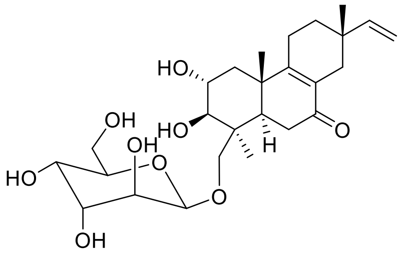<br>virescenoside M<br>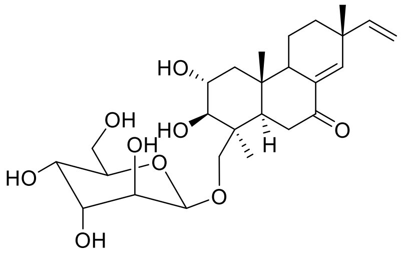<br>virescenoside V<br>or<br>their isomers	497.2745	-1.6
8	Pl<br>PlPh2	7.2	497.2737<br>$[M+H]^+$			
9	Pl<br>PlPh2	7.4	497.2737<br>$[M+H]^+$			
10	Pl	7.7	497.2737<br>$[M+H]^+$			
11	Pl<br>PlPh2	6.9	499.2909<br>$[M+H]^+$	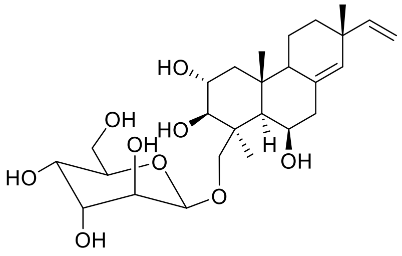<br>virescenoside N<br>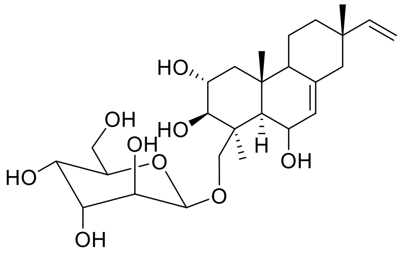<br>virescenoside W<br>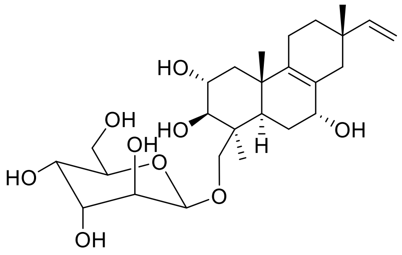<br>virescenoside X	499.2902	1.4
12	Pl	7.3	499.2909<br>$[M+H]^+$			
13	Pl<br>PlPh2	7.5	499.2909<br>$[M+H]^+$			
14	Pl<br>PlPh2	8.7	481.2803<br>$[M+H]^+$		481.2796	1.5

15	Pl<br>PlPh1<br>PlPh2	8.9	481.2803<br>[M+H]$^+$	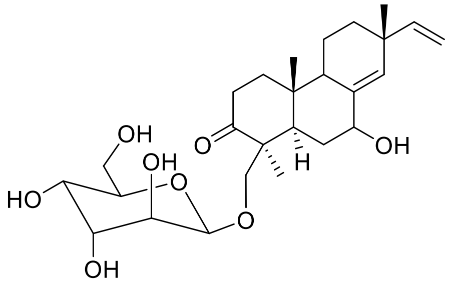<br>virescenoside S<br>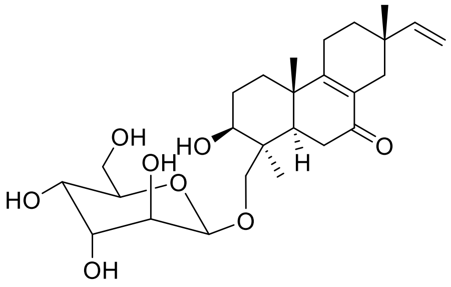<br>virescenoside P<br>or<br>their isomers		
16	Pl<br>PlPh1<br>PlPh2	9.4	481.2803<br>[M+H]$^+$			
17	Pl<br>PlPh1<br>PlPh2	10.0	481.2803<br>[M+H]$^+$			
18	Pl<br>PlPh1<br>PlPh2	11.0	481.2803<br>[M+H]$^+$			
19	Pl<br>PlPh1<br>PlPh2	9.6	645.3461<br>[M+H]$^+$	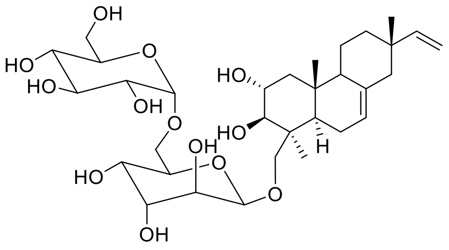<br>virescenoside R<br>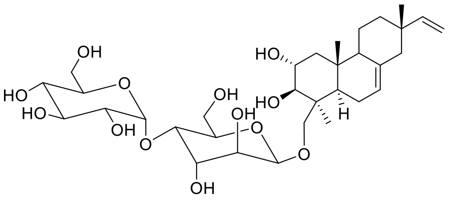<br>virescenoside R_3	645.3481	-3.1
20	Pl<br>PlPh1<br>PlPh2	10.5	645.3501<br>[M+H]$^+$			3.1
21	Pl	6.2	659.3271<br>[M+H]$^+$	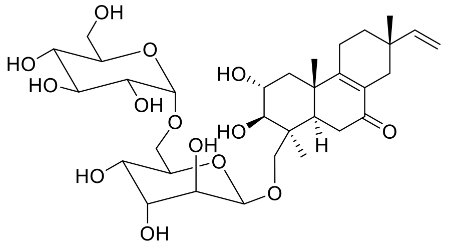<br>virescenoside R_2	659.3273	-0.3
22	Pl	6.4	479.2618<br>[M+H]$^+$	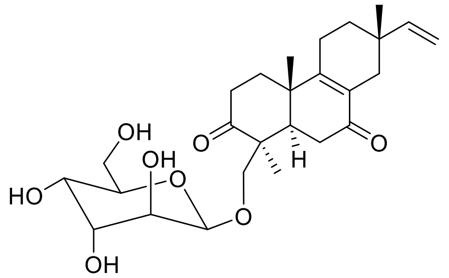	479.2639	-4.4
23	Pl	9.1	479.2618<br>[M+H]$^+$			

24	Pl<br>PlPh1	8.4	479.2653<br>[M+H]$^+$	virescenoside T<br>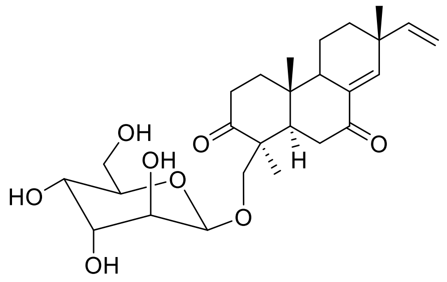<br>virescenoside U<br>or<br>their isomers		
25	Pl<br>PlPh1<br>PlPh2	9.2	479.2653<br>[M+H]$^+$		479.2639	2.9
26	Pl<br>PlPh1	9.5	479.2653<br>[M+H]$^+$			
27	Pl<br>PlPh1<br>PlPh2	16.2	1176.7974<br>[M+H]$^+$	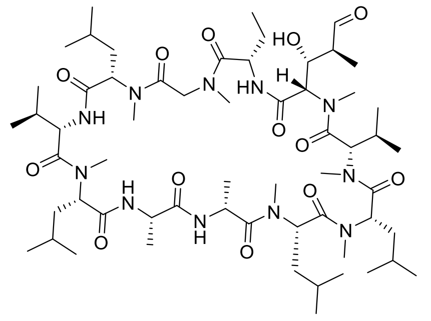<br>6-[(3R,4S)-3-Hydroxy-N-methyl-5-oxo-L-leucine]cyclosporin A<br>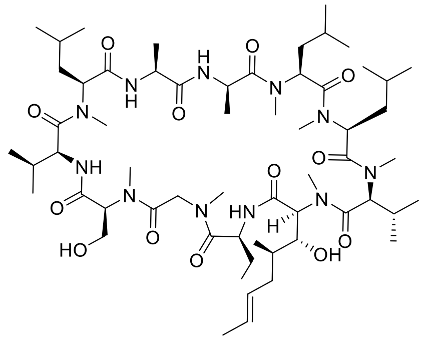<br>9-(*N*-Methyl-L-serine)cyclosporin A	1176.7966	0.7

P. hispanicum KMM4689

№	Culture	RT	m/z	Structure and name	Exact mass	Mass error
28	Ph<br>PlPh1<br>PlPh2	2.9	364.1655<br>[M+H]$^+$	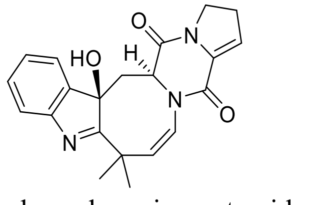<br>3β-hydroxydeoxyisoaustamide	364.1656	-0.3
29	PlPh1<br>PlPh2	4.9	209.0811<br>[M+H]$^+$	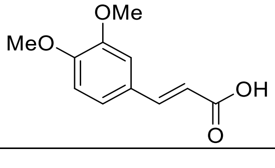	209.0804	3.3

				3,4-dimethoxycinnamic acid		
30	Ph<br>PlPh1<br>PlPh2	5.0	284.1388<br>$[M+H]^+$	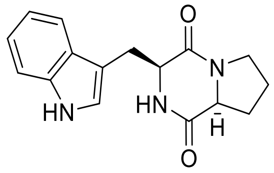<br>brevianamide F	284.1394	-2.1
31	Ph<br>PlPh1<br>PlPh2	5.1	235.0949<br>$[M+H]^+$	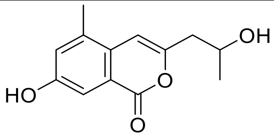<br>7-hydroxy-3-(2-hydroxypropyl)-5-methylisochromen-1-one	235.0965	-6.8
32	Ph<br>PlPh1<br>PlPh2	5.2	364.1640<br>$[M+H]^+$	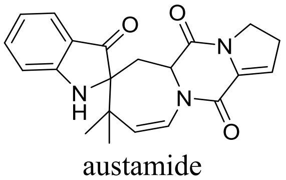<br>austamide	364.1656	-4.4
33	Ph<br>PlPh1<br>PlPh2	6.0	382.1753<br>$[M+H]^+$	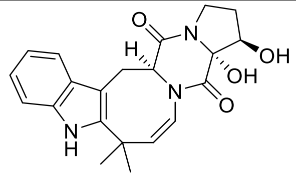<br>16β,17α-dihydroxy-deoxydihydroisoaustamide		
34	Ph<br>PlPh1<br>PlPh2	6.2	382.1753<br>$[M+H]^+$	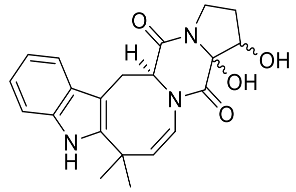<br>16,17-dihydroxy-deoxydihydroisoaustamide	382.1761	-2.1
35	Ph<br>PlPh1<br>PlPh2	6.6	382.1753<br>$[M+H]^+$	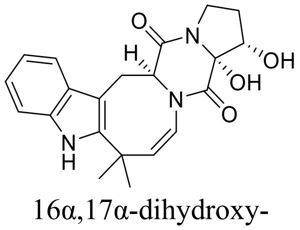<br>16α,17α-dihydroxy-deoxydihydroisoaustamide		

36	Ph<br>PlPh1<br>PlPh2	7.3	396.1895<br>$[M+H]^+$	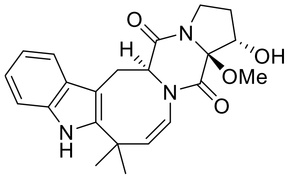<br>16α-hydroxy-17β-methoxy-deoxydihydroisoaustamide<br>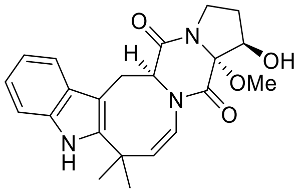<br>16β-hydroxy-17α-methoxy-deoxydihydroisoaustamide<br>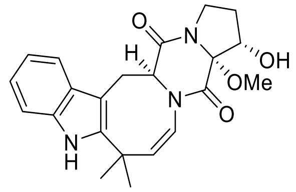<br>16α-hydroxy-17α-methoxy-deoxydihydroisoaustamide	396.1918	-5.8
37	Ph<br>PlPh1<br>PlPh2	7.6	350.1808<br>$[M+H]^+$	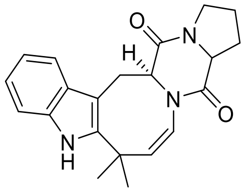<br>deoxydihydroisoaustamide	350.1863	-15.7
38	Ph<br>PlPh1<br>PlPh2	7.7	348.1713<br>$[M+H]^+$	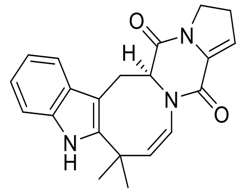<br>(+)-deoxyisoaustamide	348.1707	1.7
39	Ph<br>PlPh1<br>PlPh2	8.1	315.0488<br>$[M+H]^+$	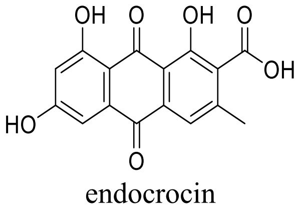<br>endocrocin	315.0499	-3.5
40	Ph<br>PlPh1<br>PlPh2	8.8	287.0539<br>$[M+H]^+$	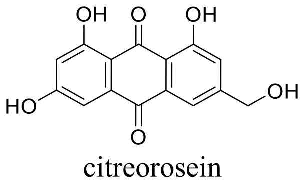<br>citreorosein	287.0550	-3.8
41	Ph<br>PlPh1<br>PlPh2	9.6	352.2019<br>$[M+H]^+$	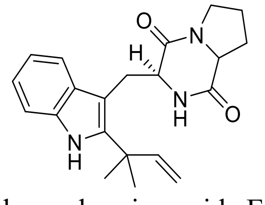<br>desoxybrevianamide E	352.2020	-0.3

42	Ph<br>PlPh1<br>PlPh2	10.2	321.0150<br>$[M+H]^+$	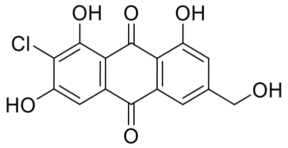<br>2-chlorocitreorosein	321.0160	-3.1
43	Ph<br>PlPh1<br>PlPh2	10.8	346.1544<br>$[M+H]^+$	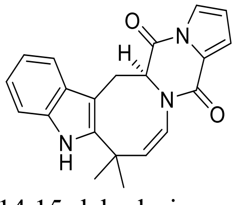<br>deoxy-14,15-dehydroisoaustamide	346.1550	-1.7
44	Ph<br>PlPh1<br>PlPh2	13.0	271.0587<br>$[M+H]^+$	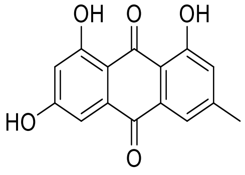<br>emodine	271.0601	-5.2
45	PlPh2	13.6	639.1697<br>$[M+H]^+$	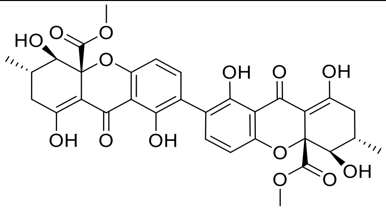<br>secalonic acid D	639.1708	-1.7
46	PlPh1<br>PlPh2	15.0	545.0999<br>$[M+H]^+$	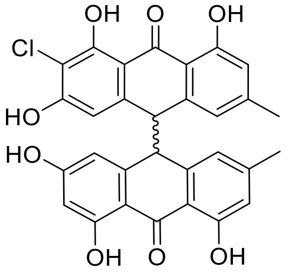<br>nephrolaevigatin D	545.0998	0.2
47	Ph<br>PlPh1<br>PlPh2	15.3	545.0999<br>$[M+H]^+$	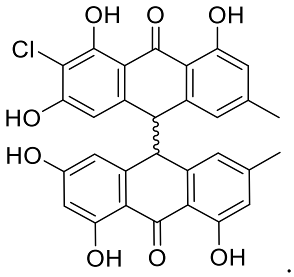<br>nephrolaevigatin C	545.0998	0.2
48	Ph<br>PlPh1<br>PlPh2	15.5	579.0665<br>$[M+H]^+$	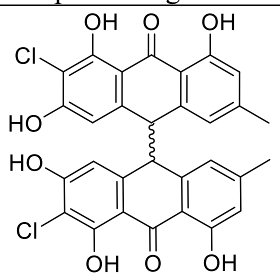<br>nephrolaevigatin A	579.0608	9.8

49	Ph<br>PlPh1<br>PlPh2	15.7	579.0626<br>$[M+H]^+$	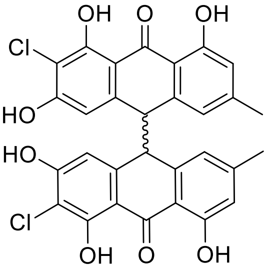<br>nephrolaevigatin B	579.0608	3.1
50	Pl<br>Ph<br>PlPh1<br>PlPh2	20.4	429.3350<br>$[M+H]^+$	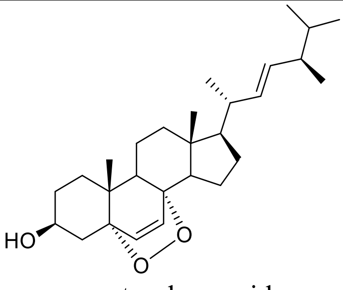<br>ergosterol peroxide	429.3363	-3.0
